# Prof. R. B. Winder

**Published:** 1894-08

**Authors:** 


### Obituary.

PROF. R. B. WINDER.—The dental profession has lost one of its most efficient laborers and brightest scholars in the death of Prof. R. B. Winder. His labors in the realm of dentistry have been incessant and valuable. He was possesed of a kindly nature, and of untiring energy. He was pre-eminent as a leader. He had few equals as an interesting lecturer; in short Prof. Winder was an ideal teacher, especially of Operative Dentistry in which he excelled, though he was very fond of Physiology also. His soul was filled with his subject, making a free use of diagrams and pictures from which his dental students got their most helpful items of knowledge. A sketch of his life follows, together with the action of the Faculty of the Baltimore College of Dental Surgery.

Dr. Richard B. Winder, dean of the Baltimore College of Dental Surgery, died at his home, 716 Park Ave., Baltimore, on July 18, 1894 of heart affection. Dr. Winder was born at Drummondtown on the Eastern Shore of Virginia, in 1828, and was educated at the University of Virginia and Princeton College, graduating from the latter in 1850. He became a planter in his native State, and at the breaking out of the war entered the Confederate army. He was a major on the staff of his cousin, General Winder. Major Winder came to Baltimore at the close of the war and studied dentistry at the Baltimore College of Dental Surgery, of which he was elected dean in 1882. He was thrice married, his last wife, who survives him, being Miss Kate Dorsey, a daughter of the late Roderick Dorsey of Frederick county. His first wives were the Misses Custis of Virginia. Dr. Winder left one son, Dr. R. Bayly Winder of Baltimore, and one daughter, Mrs. H. A. Miller of Wilmington, Del. His surviving sisters are Mrs. Charles Howard of Richmond, Va., and Mrs. George Kerr of Eastville, Va.

The funeral took place July 21, 1894. Services were held at the late residence by the Rev. Peregrine Wroth, assisted by the Revs. Arthur Chilton Powell and Wm. M. Dame. Interment in Loudon Park. The pall-bearers were: Active—Dr. Thomas A. Latimer, Dr. William F. Lockwood, Dr. Pembroke Lee Thom, Mr. Charles H. Cowman, Dr. M. W. Foster, Dr. William B. Finney and Dr. B. Holly Smith. Honorary pall-bearers—Judge D. Girard Wright, William Henry Baldwin, Jr., William Floyd, John S. Hayes, Henry Coudon, Willoughby N. Smith, Dr. Edward Nelson, Dr. William Worter, Dr. George Preston and Dr. Opie.

The Faculty of the Baltimore College of Dental Surgery made the following minute:

As an executive officer he had no superior. His energy was unflagging, his intelligence unerring and his devotion to the duties of his office and to the cause of dental education was most exemplary. Therefore, it is

Resolved, That in his death the College has sustained an irreparable loss.

That the members of the Faculty mourn the death of one who was in life conspicuous for his cordiality, his active recognition of the obligations of friendship and for his high social attainments.

That a copy of these resolutions be transmitted to his family, to whom in their sorrow we tender our heartfelt sympathy.

